# Epigenetic regulation of CDH1 exon 8 alternative splicing in gastric cancer

**DOI:** 10.1186/s12885-015-1983-5

**Published:** 2015-12-16

**Authors:** Xiao-Wei Li, Bing-Yu Shi, Qing-Lan Yang, Jie Wu, Hui-Min Wu, Yu-Feng Wang, Zhi-Jiao Wu, Yi-Mei Fan, Ya-Ping Wang

**Affiliations:** Department of Medical Genetics, Jiangsu Key Laboratory of Molecular Medicine, Medical School, Nanjing University, Hankou Road 22, Nanjing, 210093 China

**Keywords:** Alternative splicing, CDH1, Histone modifications, DNA methylation, Gastric cancer

## Abstract

**Background:**

The tumor suppressor gene *CDH1* is critical for intercellular adhesion. In our previous work, we reported a nonfunctional CDH1 transcript that lacks the final 83 base pairs of exon 8 (1054*del*83). In this work, we probed the role of histone epigenetic modifications as well as DNA methylation in selection of this isoform.

**Methods:**

RT-qPCR was used to detect *CDH1* RNA expression. Methylation of *CDH1* was analyzed by bisulphite sequencing PCR. ChIP assay was performed to show histones level. Cell lines were treated with DNA methyltransferase inhibitor AZA, HDAC inhibitor TSA, or siRNA oligonucleotides to test regulation of CDH1 splicing.

**Results:**

Greater CDH1 1054*del*83 transcripts were observed in gastric cancer (GC) cell lines than human gastric mucosal epithelial cell line GES-1. All the cell lines showed significant methylation pattern at the CpG sites of CDH1 exon 8. AZA treatment did not influence selection of 1054*del*83 transcripts. A significant decrease in acetylation for histones H3 and H4K16Ac in an internal region of the CDH1 gene surrounding the alternative exon 8 were detected in GC cell lines. Treatment with TSA preferentially expressed the correctly spliced transcript and not the exon 8 skipped aberrant transcripts, showing that histone acetylation was involved in the splicing regulation. SiRNA-mediated knockdown of SETD2 (The specific methyltransferase of H3K36) decreased exclusion of exon 8, suggesting that the presence of this mark correlates with increased skipping of the final 83 base pairs of CDH1 exon 8. However, CDH1 splicing was not affected by SRSF2 knockdown.

**Conclusions:**

H3K36me3 correlates with increased skipping of the final 83 base pairs of CDH1 exon 8. Histone acetylation was involved in the splicing regulation as well.

**Electronic supplementary material:**

The online version of this article (doi:10.1186/s12885-015-1983-5) contains supplementary material, which is available to authorized users.

## Background

Gastric cancer (GC) is one of the most common malignancies worldwide, with the highest incidence rates in Eastern Asia [1]. It is believed that GC is a multistep process during which some genetic alterations such as oncogene activation, tumor suppressor gene inactivation and DNA repair deficiency are responsible for the overall outcome of the cancer.

The tumor suppressor gene *CDH1* (E-cadherin) is critical for intercellular adhesion [2, 3]. *CDH1* gene mutations occurred frequently in hereditary diffuse gastric cancer (HDGC) [4, 5]. In our previous work in GC patients, we identified several germline mutations in *CDH1* gene [6, 7].

Most human genes are alternatively spliced in a cell type–and tissue-specific manner, and abnormalities of pre-mRNA alternative splicing contribute to disease. In our previous work, we reported an alternatively spliced, nonfunctional CDH1 transcript that lacks the final 83 base pairs of exon 8 (1054*del*83) of the gene. This non-functional transcript has a premature termination codon (PTC) with 358 aminos and is degraded by the nonsense mediated decay (NMD) pathway. We demonstrated this transcript is a frequent event in Chinese GC patients [7].

Analysis of alternative splicing regulation has traditionally focused on RNA sequence elements and their associated splicing factors [8–11]. But 1054*del*83 transcript seemed to be not triggered by RNA sequence elements [7]. Research disclosed that pre-mRNA splicing generally proceeds cotranscriptionally [12], and thus give the basis of epigenetic regulation of alternative splicing. Recent studies provided evidence that alternative splice site choice is influenced by chromatin structure and histone modifications mainly through two mechanisms, kinetic coupling [13–18] or chromatin-splicing adaptor systems [13, 19–22]. Given these observations, we probed the role of histone epigenetic modifications as well as DNA methylation in pre-mRNA alternative splicing of CDH1 exon 8.

## Methods

### Cell culture

The GC cell lines SGC-7901, BGC-823 and MGC80-3 (Purchased from Shanghai Cell Bank of Chinese Academy of Sciences, China) and human gastric mucosal epithelial cell line GES-1 (Purchased from Cell bank of Xiangya Medical School, Central South University, China) were cultured in DMEM medium, supplemented with 10 % fetal calf serum, at 37 °C with 5 % CO_2._ Emetine treatment was done at concentration of 100 μg/ml for 8 h before harvest of the cells. The three GC cell lines were poorly differentiated adenocarcinoma cells. No ethics approval was required for this study.

### RNA extraction and quantitative reverse transcription polymerase chain reaction (RT-qPCR)

Total RNA from GC cell lines or human gastric mucosal epithelial cell line was extracted using RNAiso Plus (TaKaRa Biotechnology (Dalian) Co., Ltd.). RT–qPCR was performed in two steps. First strand cDNA synthesis was performed using PrimerScript RT reagent Kit (TaKaRa) with random DNA hexamers and oligo-dT primer according to the manufacturer’s protocol. CDH1 expression is analyzed by a TaqMan qPCR experiment on ABI StepOne Plus Real-Time PCR System (Applied Biosystems, Foster City, CA, USA). The qPCR reaction condition and sequences of PCR primers and TaqMan probes are as previous reported [7].

### DNA methylation assay

DNA was treated with bisulfite using the CpGenome™ DNA Modification Kit (CHEMICON International, Temecula, CA, USA) according to manufacturer’s protocol. Methylation status of CpG sites of *CDH1* exon 7–9 was analyzed by bisulphite sequencing PCR (BSP) on an ABI 3130-Avant automated sequencer (Applied Biosystems). Primer sequences for BSP are *CDH1*-E7-BSP-F:5′-TGAATTTTTTTAGGAATTTTTGTGAT-3′, *CDH1*-E7-BSP-R:5′-ATCCAACCCAATAATAACCACACTA-3′, CDH1-E8-BSP-F: 5′-GGGTTAGGTTAAAGGTGGTTAGTGT-3′, CDH1-E8-BSP-R: 5′-AAACCTTTCTTTAAAAACCCTCTAAAA-3′, *CDH1*-E9-BSP-F:5′-AGTATAAGGGTTAGGTGTTTGAGAA-3′ and *CDH1*-E9-BSP-R:5′-CTACATCTTACCAAATACCATACAAACC-3′.

### Chromatin immunoprecipitation (ChIP) assay

ChIP assays were performed by using the EZ-Magna ChIP Kit (catalog no.17-408; Millipore, USA) according to the manufacturer’s instructions. Briefly, 1 × 10^6^ cells were fixed with 1 % formaldehyde for 10 min at 37 °C. The cells were washed extensively with PBS, and the chromatin was sheared by sonication (Bioruptor sonicator) to 200–500 bp fragments. The cross-linked histone-DNA complex was immunoprecipitated with anti-H3K36me3, anti-H3K4me2, anti-H4K16ac, or anti-pan acetylated-H3 (anti-acH3) antibodies (Millipore). Normal rabbit IgG was used as negative controls. DNA was obtained from the crosslinked complex and equal amounts of input and immunoprecipitated DNA 1.0 ng were used to perform SYBR Green real-time PCR on ABI StepOne Plus Real-Time PCR System (Applied Biosystems). The qPCR reaction mixture contained DNA, the forward primer, reverse primer, ROX Reference Dye, SYBR and Premix Ex Taq™ (TaKaRa). Primers are described in Additional file 1: Table S1. The thermal cycle conditions for assay were as follows: 95 °C at 30 sec, 40 cycles at 95 °C for 15 sec and 60 °C for 30 sec.

### CDH1 expression with DNA methyltransferase inhibitor AZA or histone deacetylases (HDAC) inhibitor trichostatin A (TSA)

To test whether CDH1 splicing is affected by DNA methylation or histone acetylation status, the GC cell lines SGC-7901, BGC-823 and MGC80-3 and human gastric mucosal epithelial cell line GES-1 were treated with DNA methyltransferase inhibitor AZA or HDAC inhibitor TSA. 5-aza-2′deoxycytidine (Sigma-Aldrich) was added to the medium to be 1 μM. Treatments were maintained for 72 h. TSA (Sigma-Aldrich) was added to culture to be 0.5 μM 12 h before the end of the experiment. CDH1 RNA expression was determined by RT-qPCR analysis.

### siRNA-mediated transient knockdown

Downregulation of SETD2 or SRSF2 was performed using siRNA oligonucleotides (Guangzhou Ribobio Co., LTD, China). SiRNA oligos against human SETD2 or SRSF2 were delivered to cells at 50 nM following the manufacturer’s instructions. Normal negative control was used as control. Forty eight hours after transfections, cells were harvested and the knockdown efficiencies were analyzed by RT-qPCR and changes in CDH1 splicing were analyzed as mentioned above.

### Comparative in silico analysis

NNSPLICE (http://www.fruitfly.org/seq_tools/splice.html) were used to predict splice acceptors and donors around *CDH1* exon 8. We used the ESEfinder program (http://rulai.cshl.edu/tools/ESE) to identify exonic splicing enhancers (ESEs) [23].

### Statistical analysis

Differences in transcripts level between groups were analyzed by ANOVA (analysis of variance) and SNK-q test. All *P* values are two-sided; *P <* 0.05 was considered statistically significant.

## Result

### Two alternative donor sites were in CDH1 exon 8 and flanking sequences

**Fig. 1:**
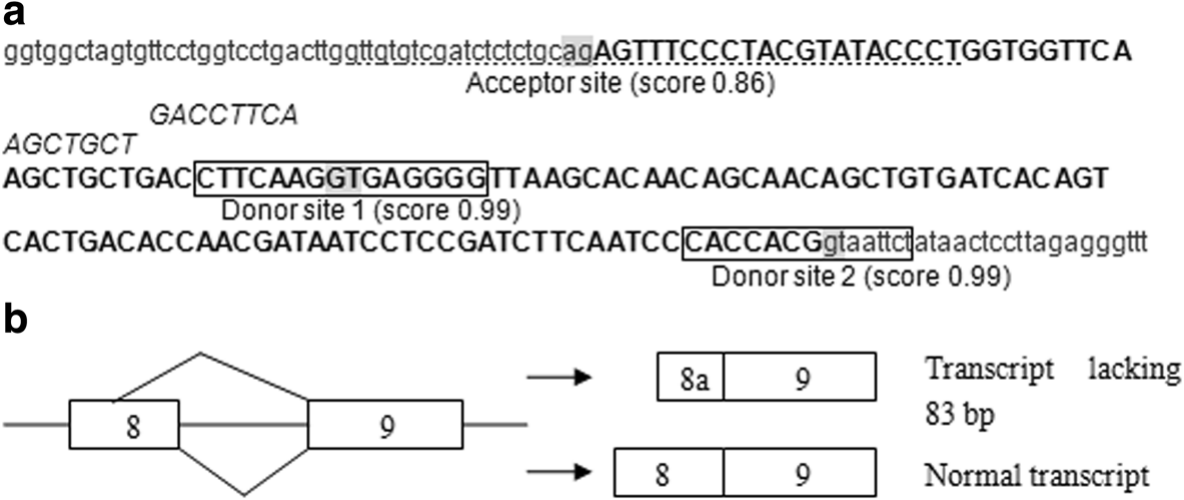
**a** Splice sites prediction by bioinformatic analysis*.* Capital letters show CDH1 exon 8 and lowercases, the flanking intron sequences. The italic characters indicate SRSF2 motifs flanking donor site 1. **b** Schematic diagram of alternative splicing of CDH1 exon 8

We find two donor sites in *CDH1* exon 8 and flanking sequences which will produce the 1054*del*83 transcript and normal transcript, respectively [7] (Fig. 1). ESEfinder predicted that there are two extra SRSF2 motifs flanking donor site 1 (Fig. 1a).

### GC cells carry significant more CDH1 1054del83 isoform than GES-1 cells

**Fig. 2:**
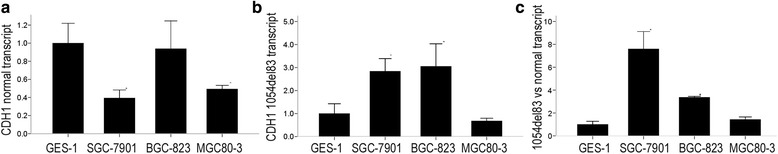
Expression of CDH1 normal transcript (**a**), CDH1 1054*del*83 transcript (**b**), and ratio of expression value of CDH1 1054*del*83 transcript vs CDH1 normal transcript (**c**) in human gastric mucosal epithelial cell line GES-1 and the GC cell lines SGC-7901, BGC-823 and MGC80-3. The expression level in GES-1 was set to 1. The star * means *P <* 0.05, indicating statistically significant

RT-qPCR revealed the coexistence of the normal CDH1 transcript and CDH1 1054*del*83 transcript in the GC cell lines SGC-7901, BGC-823 and MGC80-3 and human gastric mucosal epithelial cell line GES-1. A lower expression of the normal CDH1 and a higher level of CDH1 1054*del*83 transcript are detected in GC cell lines SGC-7901 and BGC-823 compared to GES-1 (Fig. 2a and b). Taken together, there is a higher ratio of CDH1 1054*del*83 vs CDH1 normal transcript in GC cell lines SGC-7901 and BGC-823 than in GES-1 (Fig. 2c). However, in GC cell line MGC80-3, though a lower expression of the normal CDH1 was detected (Fig. 2a), no difference existed for level of CDH1 1054*del*83 transcript between MGC80-3 and GES-1 (Fig. 2b and c). So, in most of the experiments carried out behind, we used GC cell lines SGC-7901 and BGC-823 for comparing splicing with GES-1.

### Hypermethylation was shown at the CpG sites of CDH1 exon 8 and the nearby exons

**Fig. 3:**
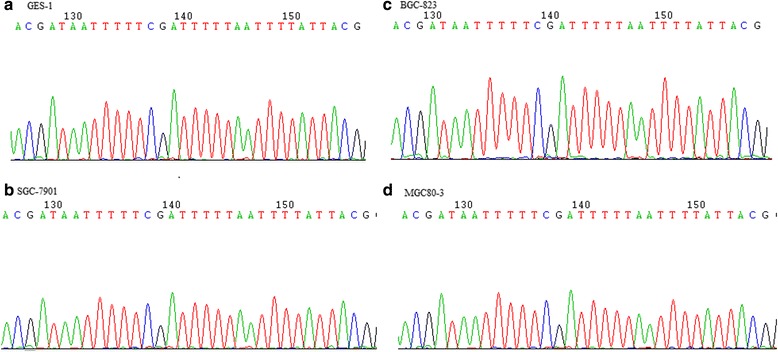
Methylation status of CDH1 exon 8 in human gastric mucosal epithelial cell line GES-1 (**a**) and the GC cell lines SGC-7901 (**b**), BGC-823 (**c**) and MGC80-3 (**d**). DNA isolated from cells shows a high C content at all CpGs attributable to reduced bisulfite modification because of complete methylation of the DNA

All the three GC cell lines SGC-7901, BGC-823 and MGC80-3, and the human gastric mucosal epithelial cell line GES-1 showed significant methylation pattern at the CpG sites of CDH1 exon 8 and the nearby exons (Fig. 3, Additional file 2: Figure S1 and Additional file 3: Figure S2).

### Lower level of histone acetylation and higher level of H3K36 tri-methylation were detected around CDH1 exon 8 regions in GC cell lines compared to GES-1

**Fig. 4:**
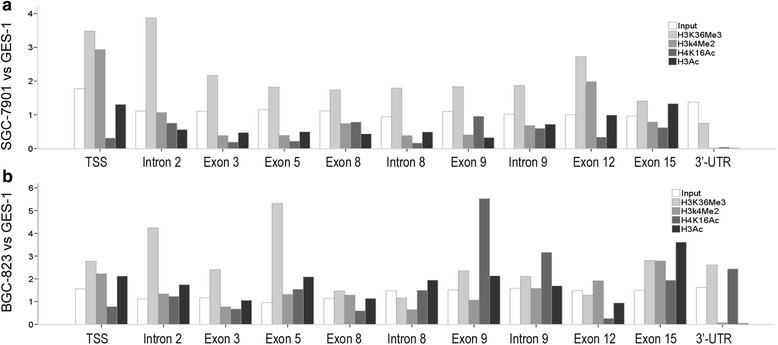
Histone modification patterns of CDH1 in SGC-7901 (**a**) and BGC-823 (**b**) compared to GES-1. The graphs demonstrate fold differences of distinct histone modifications at different region of CDH1 between SGC-7901 or BGC-823 and GES-1 cells

To get a picture of the distribution of histone modifications across the CDH1 gene, we performed ChIP assay. We first performed ChIP of H3 acetylation and H4K16Ac in extracts from cells. ChIP results showed a significant decrease in acetylation for histones H3 in an internal region of the CDH1 gene surrounding the alternative exon 8 in SGC-7901 cells compared to human gastric mucosal epithelial cell line GES-1. A lower H4K16Ac were detected in this region both in SGC-7901 and BGC-823 cells compared to GES-1 (Fig. 4).

To investigate if CDH1 exon 8 splicing could be modulated by a change in the histone methylation patterns, we performed ChIP studies using antibodies recognizing methylation at different lysines of histone H3. No regular difference of H3K4Me2 was detected between GC cell lines and human gastric mucosal epithelial cell line GES-1 (Fig. 4). To the contrary, analysis of H3K36 tri-methylation revealed higher level of H3K36 tri-methylation around the CDH1 exon 8 regions in SGC-7901 and BGC-823 compared to GES-1 (Fig. 4).

### TSA treatment of cells led to a significant change in splicing in favor of CDH1 normal transcript, while AZA treatment did not affect the amount of CDH1 full length and CDH1 1054del83 splice isoform

**Fig. 5:**
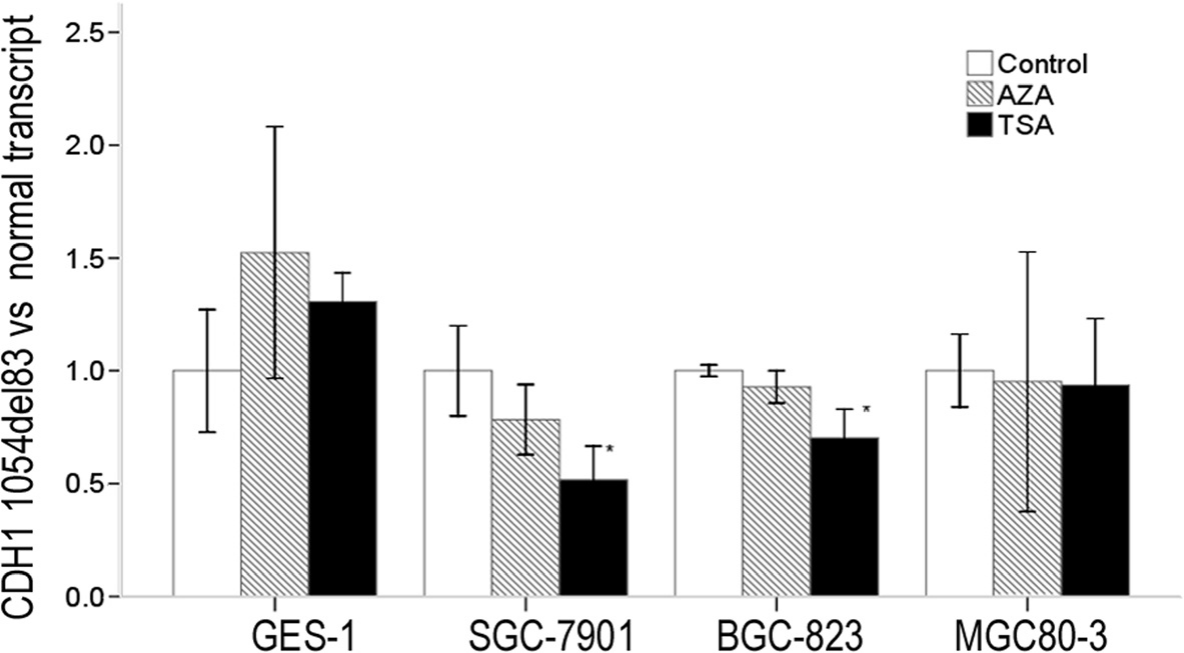
The relative folds of ratios of CDH1 1054*del*83 transcript vs CDH1 normal transcript after AZA or TSA treatment. The values were calculated as (2^-ΔCt (1054del83 transcript-actin)^/2^-ΔCt (normal transcript-actin)^). Relative folds of ratios of untreated cell lines serve as 1. The star * means *P <* 0.05, indicating statistically significant

**Fig. 6:**
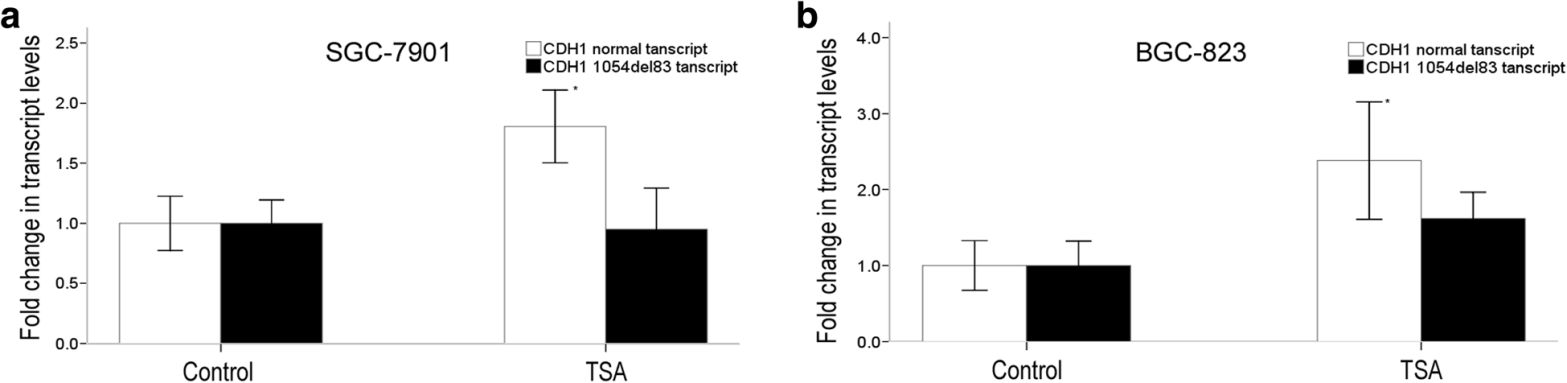
The level change of CDH1 normal transcript and CDH1 1054*del*83 transcript after TSA treatment in SGC-7901 (**a**) and BGC-823 (**b**). The values were calculated as 2^-ΔCt (1054del83 transcript-actin)^ or 2^-ΔCt (normal transcript-actin)^. The values of untreated cell lines were set to 1. The star * means *P <* 0.05, indicating statistically significant

To explore a putative regulation of the alternative splicing of CDH1 exon 8 by means of histone acetylation or DNA methylation, we treated the GC cell lines SGC-7901, BGC-823, and MGC80-3 and human gastric mucosal epithelial cell line GES-1 with HDAC inhibitor TSA or DNA methyltransferase inhibitor AZA. As shown in Fig. 5, AZA treatment did not influence ratios of CDH1 1054*del*83 transcript vs CDH1 normal transcript in all the four cell lines. TSA deceased ratios of CDH1 1054*del*83 transcript vs CDH1 normal transcript in the two GC cell lines SGC-7901 and BGC-823. Detail analysis showed after TSA treatment, CDH1 normal transcript incerased to about 2 fold, while the CDH1 1054*del*83 transcript did not change significantly (Fig. 6).

### siSETD treatment showed a shift in the splicing of the CDH1 pre-mRNAs in favor of CDH1 normal transcript

**Fig. 7:**
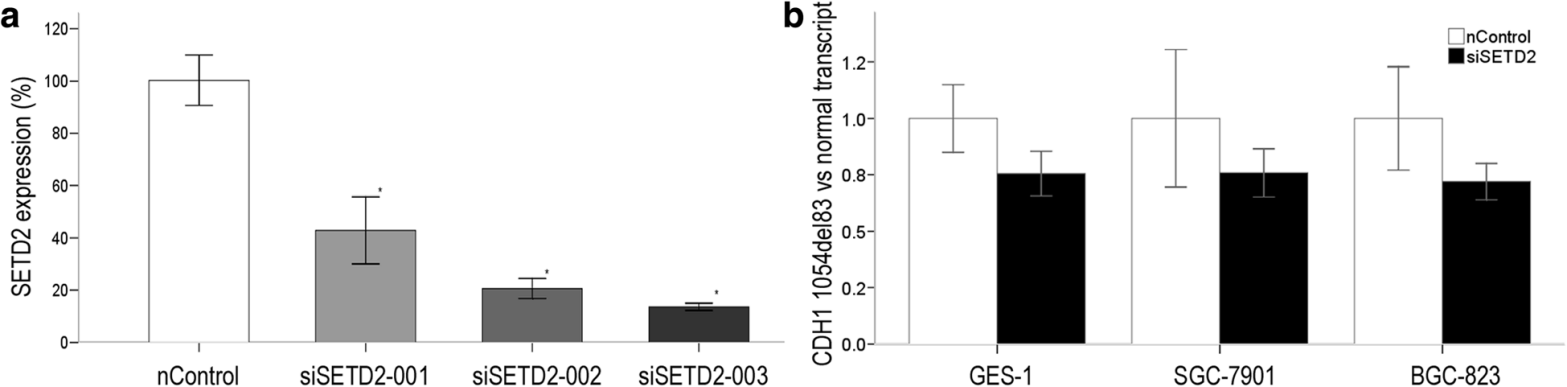
CDH1 expression after siSETD2 treatment. **a** Interference efficiency of the three kinds of siSETD2 in GES-1 cells. The values were calculated as 2^-ΔCt (SETD2-actin)^. The star * means *P <* 0.05, indicating statistically significant. **b** The relative folds of ratios of CDH1 1054*del*83 transcript vs CDH1 normal transcript after siSETD2-003 treatment. The values were calculated as (2^-ΔCt (1054del83 transcript-actin)^/2^-ΔCt (normal transcript-actin)^). Relative folds of ratios of untreated cell lines serve as 1

A value of nearly 90 % SETD2 knockdown was determined by qPCR analysis after siSETD2-003 treatment of GC cell lines and human gastric mucosal epithelial cell line (Fig. 7a and Additional file 4: Figure S3). Decreased ratios of CDH1 1054*del*83 transcript vs CDH1 normal transcript was observed in all the three cell lines transiently transfected with SETD2 siRNA compared to cells transfected with scramble (nontargeting) siRNA (Fig. 7b).

### Knockdown of SRSF2 did not influence ratios of CDH1 alternative transcripts

**Fig. 8:**
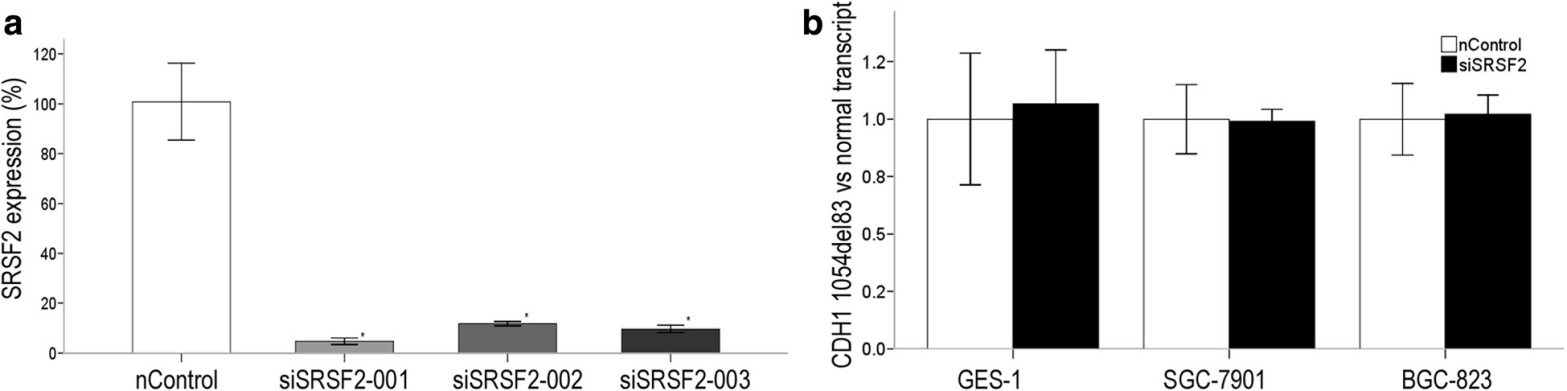
CDH1 expression after siSRSF2 treatment. **a** Interference efficiency of the three kinds of siSRSF2 in GES-1 cells. The values were calculated as 2^-ΔCt (SRSF2-actin)^. The star * means *P <* 0.05, indicating statistically significant. **b** The relative folds of ratios of CDH1 1054*del*83 transcript vs CDH1 normal transcript after siSRSF2-001 treatment. The values were calculated as (2^-ΔCt (1054del83 transcript-actin)^/2^-ΔCt (normal transcript-actin)^). Relative folds of ratios of untreated cell lines serve as 1

The interference efficiency of the three kinds of siSRSF2 can all get about 90 % for SRSF2 expression (Fig. 8a and Additional file 5: Figure S4). SiSRSF2 treatment did not influence ratios of CDH1 1054*del*83 transcript vs CDH1 normal transcript in all the three kinds of cells (Fig. 8b).

## Discussion

The *CDH1* gene, a calcium-dependent transmembrane glycoprotein, is critical for epithelial architecture and intercellular adhesion. Sharma et al. have demonstrated the existence of transcripts with CDH1 exon 11 skipping in chronic lymphocytic leukemia cells and head and neck cancer cells [24, 25]. Further analysis showed a low histone acetylating level of CDH1 exon 11 in chronic lymphocytic leukemia cells. HDAC inhibitors MS-275 treatment increased the level of normal CDH1 transcript [26].

The 1054*del*83 transcript had been reported in HDGC [27]. Our previous study demonstrated this aberrant transcript existed in GC patients harboring no mutations, which suggested it could be a frequent event in GC patients [7]. In this study, we further show the skipping is not a specific feature of GC, since these transcripts occurred in GC cell lines and the human gastric mucosal epithelial cell line GES-1 as well. However, GC cells carry significant more CDH1 1054*del*83 isoform than GES-1 cells (Fig. 2).

The CDH1 1054*del*83 isoform was suggested to move the reading frame and create a PTC with 358 aminos, which would presumably lead to nonsense mediated mRNA decay. CDH1 1054*del*83 will serve to down-regulate the amount of full-length CDH1 mRNA/protein produced and lead to reduction of CDH1 activity. We might suggest the increase of CDH1 1054*del*83 isoform might lead to GC.

There might be a natural balance of the two alternative splice products (normal CDH1 and 1054*del*83 transcripts). To explore a putative regulation of the alternative splicing of CDH1 exon 8 by means of epigenetic, we carried out in vitro experiment.

*Shukla S* has reported DNA methylation could regulate alternative splicing in *CD45* exon5 [18]. In our data, there is no correlation between DNA methylation and CDH1 exon 8 alternative splicing (Fig. 5). The influence of DNA methylation modifications on exon skipping might be critical in some genes but not in others.

In the period of transcription, transcriptional elongation speed is modulated by the dynamic balance of acetylation and deacetylation of histones. Recent researches have indicated that local histone acetylation patterns influence splice site selection [14–17].

In our study, a significant decrease in acetylation for histones H3 and H4K16Ac in an internal region of the CDH1 gene surrounding the alternative exon 8 were detected in GC cell lines. Treatment with TSA preferentially expressed the correctly spliced transcript and not the exon 8 skipped aberrant transcripts. A derived hypothesis would be that low level of histone acetylation in GC cells would cause a more compact chromatin structure, thus slower transcriptional elongation speed of Pol II and more time for suboptimal splicing signal (donor site 1) to be recognized by the splicing machinery, and thus the CDH1 1054*del*83 transcript is enhanced. Inhibition of HDAC activity with TSA will increase acetylation of H3 and H4 and induce chromatin opening and faster rate of transcriptional elongation, decreasing the use of donor site 1 and thus less the 1054*del*83 transcript.

Analysis of histone methylation revealed an increase for H3K36 tri-methylation surrounding the CDH1 exon 8 regions in GC cell lines SGC-7901 and BGC-823 compared to human gastric mucosal epithelial cell line GES-1 (Fig. 4). Down-regulation of the H3-K36 methyltransferase SETD2 by RNA interference showed a shift in the splicing of the CDH1 pre-mRNAs in favor of CDH1 normal transcript (Fig. 7). These results demonstrate that histone modifications H3K36 tri-methylation can enhance the use of donor site 1. The presence of this mark correlates with increased exclusion of the final 83 base pairs of CDH1 exon 8 in the mature CDH1 mRNA. Our results demonstrate a role for H3K36 tri-methylation in alternative splicing control. But how does it work? Physical interaction between several chromatin-associated proteins and splicing components has been reported, which have been elucidated as chromatin-splicing adaptor systems [13, 19–22]. ESEfinder predicted that compared to donor site 2 of CDH1 exon 8, there are two extra ESE motifs flanking donor site 1 region which can be bind by SRSF2, a sequence-specific RNA binding factor that promotes spliceosome formation (Fig. 1). We propose that the H3K36me3 mark might be recognized by chromatin remodeling proteins, which directly recruits splicing factors SRSF2 to the exonic splicing enhancer element surrounding donor site 1 of CDH1 exon 8 to increase 1054*del*83 transcript. However, knockdown of SRSF2 did not influence ratios of CDH1 1054*del*83 transcript vs CDH1 normal transcript in the GC cell lines and GES-1 cell line (Fig. 8). Thus, we could not get a proof for the effect of SRSF2 on splicing of CDH1 pre-mRNA. These results indicate that H3K36 tri-methylation play a role in CDH1 splice site selection, but the recruitment of chromatin remodeling proteins and splicing factors is more complicated than predicted. Additional studies are needed to disclose the mechanism how this happens.

Though GC cell lines SGC-7901 and BGC-823 carry significant more CDH1 1054*del*83 isoform than GES-1 cell line, the difference did not exist between GC cell lines MGC80-3 and GES-1 (Fig. 2). The HDAC inhibitor TSA led to a significant change in splicing in favor of CDH1 normal transcript both in the two GC cell lines SGC-7901 and BGC-823, but not in GC cell line MGC80-3 (Fig. 5). The influence of epigenetic modifications on exon skipping might be critical in some cells but not in others.

## Conclusions

The chromosomal region encompassing the CDH1 exon 8 is highly enriched in H3K36me3 marks in GC cells compared to human gastric mucosal epithelial cells, and the presence of this mark correlates with increased skipping of the final 83 base pairs of CDH1 exon 8 in the mature CDH1 mRNA. We propose that the epigenetic modification patterns, such as histone acetylation might have a role in CDH1 exon 8 alternative splicing regulations as well. The linking between histone modifications and splicing regulation might be important in GC occurrence.

## Additional files

Additional file 1: Table S1. Sequences of primers used in CDH1 ChIP assay. (DOC 32 kb) Additional file 2: Figure S1. Methylation status of CDH1 exon 7 in human gastric mucosal epithelial cell line GES-1 and the GC cell lines SGC-7901, BGC-823 and MGC80-3. DNA isolated from cells shows a high C content at all CpGs attributable to reduced bisulfite modification because of partially methylation of the DNA. (TIF 1249 kb) Additional file 3: Figure S2. Methylation status of CDH1 exon 9 in human gastric mucosal epithelial cell line GES-1 and the GC cell lines SGC-7901, BGC-823 and MGC80-3. DNA isolated from cells shows a high C content at all CpGs attributable to reduced bisulfite modification because of nearly complete methylation of the DNA. (TIF 1354 kb) Additional file 4: Figure S3. Interference efficiency of the three kinds of siSETD2 in SGC-7901 (**A**) and BGC-823 (**B**) cells. The values were calculated as 2^-ΔCt (SETD2-actin)^. The star * means *P <* 0.05, indicating statistically significant. (TIF 147 kb) Additional file 5: Figure S4. Interference efficiency of the three kinds of siSRSF2 in SGC-7901 (**A**) and BGC-823 (**B**) cells. The values were calculated as 2^-ΔCt (SRSF2-actin)^. The star * means *P <* 0.05, indicating statistically significant. (TIF 147 kb)

## Abbreviations

anti-acH3: anti-pan acetylated-H3
BSP: bisulphite sequencing PCR
ChIP: chromatin immunoprecipitation
ESEs: exonic splicing enhancers
GC: gastric cancer
HDAC: histone deacetylases
HDGC: hereditary diffuse gastric cancer
NMD: nonsense mediated decay
PTC: premature termination codon
RT-qPCR: quantitative reverse transcription polymerase chain reaction
TSA: trichostatin A
