# Supplementary material for: Epigenetic regulation of CDH1 exon 8 alternative splicing in gastric cancer
Source: BMC Cancer. 2015 Dec 16;15:954. doi: 10.1186/s12885-015-1983-5 (PMC4682244; doi:10.1186/s12885-015-1983-5)
Supplement: Additional file 1: Table S1. — Sequences of primers used in CDH1 ChIP assay. (DOC 32 kb) [file 12885_2015_1983_MOESM1_ESM.doc]

**Additional file 1: Table S1**. Sequences of primers used in CDH1 ChIP assay.

| Location | Forward Primer (5’-3’) | Reverse Primer (5’-3’) | Amplicon size (bp) |
| --- | --- | --- | --- |
| TSS | CGGGCCGTCAGCTCCGCCCTGGGGA | GAGGCACCGCCCCCCGTACCGCTGA | 100 |
| Intron2 | TATCAAGACAGGTGAAGGGAAATG | GCTGGTGCTTGTTACACACC | 105 |
| Exon3 | CCTCGACACCCGATTCAAAGTG | GAGTCCCAGGCGTAGACCAAGA | 105 |
| Exon5 | GAAGGCAAGGTTTTCTACAGCA | CTCTGTCACCTTCAGCCATCCT | 102 |
| Exon8 | CTGGTGGTTCAAGCTGCTGA | ATCGGAGGATTATCGTTGGTGT | 95 |
| Intron8 | ACCTGGTTTCATTTCTTTAT | ATGAGAATGGAATCTTTGTG | 101 |
| Exon9 | TGAATGATGATGGTGGACAA | CCAGTTTCTGCATCTTGCCAGGTAC | 108 |
| Intron9 | CTACTAGGGAGGCTGAGGCAGGAGA | CTGTCACCCAGAGCTGGAGTGCAAT | 98 |
| Exon12 | TCTGCTGATCCTGTCTGATGTGAATG | AATGATGTTTATGACCTGAGGCTTT | 103 |
| Exon15 | TAACGACGTTGCACCAACCCTC | GTGGATTACTTACTTCATCAATAAA | 101 |
| 3’-UTR | AGAATAGTGCCTAAAGTGCTGC | AGACCCACCTCAATCATCCTCA | 132 |
