# Supplementary figures and images for: Epigenetic regulation of CDH1 exon 8 alternative splicing in gastric cancer
Source: BMC Cancer. 2015 Dec 16;15:954. doi: 10.1186/s12885-015-1983-5 (PMC4682244; doi:10.1186/s12885-015-1983-5)

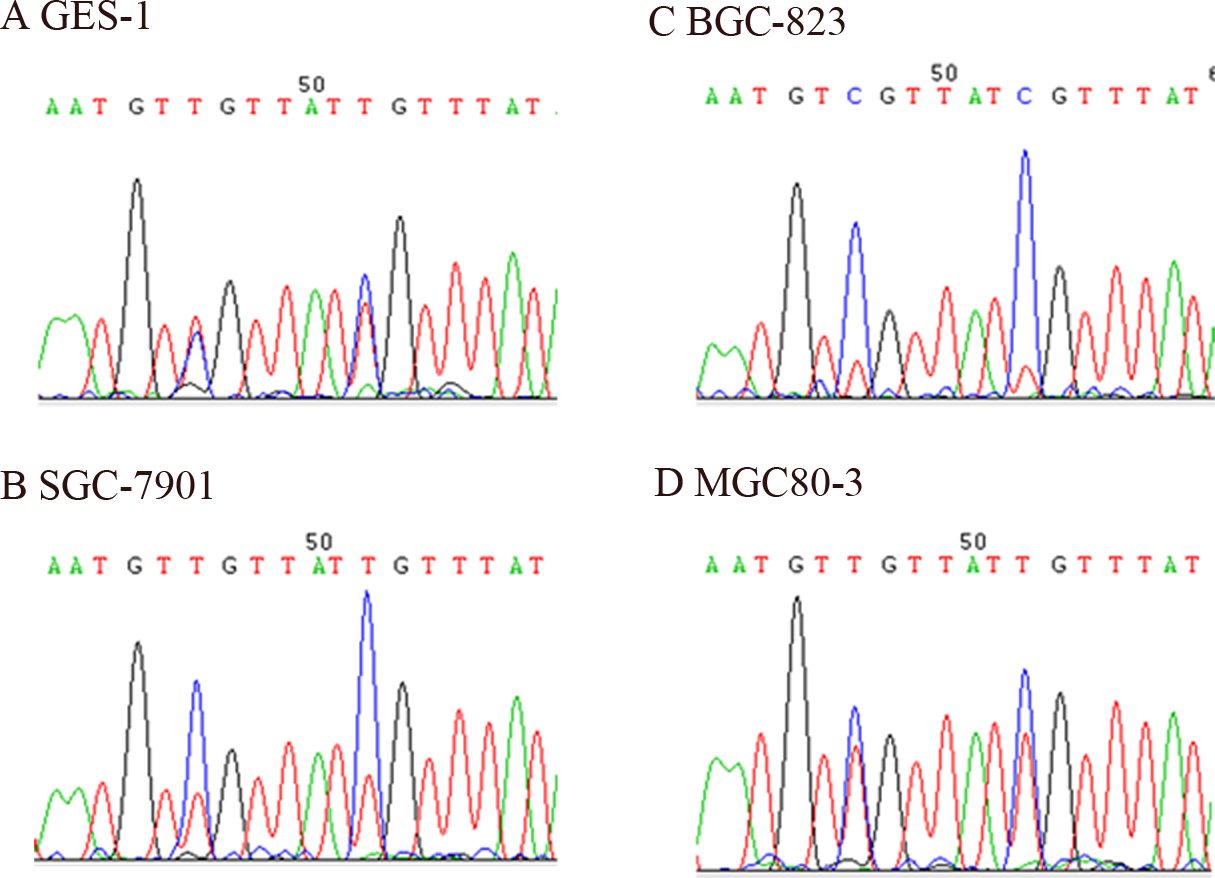

Supplement: Additional file 2: Figure S1. — Methylation status of CDH1 exon 7 in human gastric mucosal epithelial cell line GES-1 and the GC cell lines SGC-7901, BGC-823 and MGC80-3. DNA isolated from cells shows a high C content at all CpGs attributable to reduced bisulfite modification because of partially methylation of the DNA. (TIF 1249 kb) [file 12885_2015_1983_MOESM2_ESM.tif]

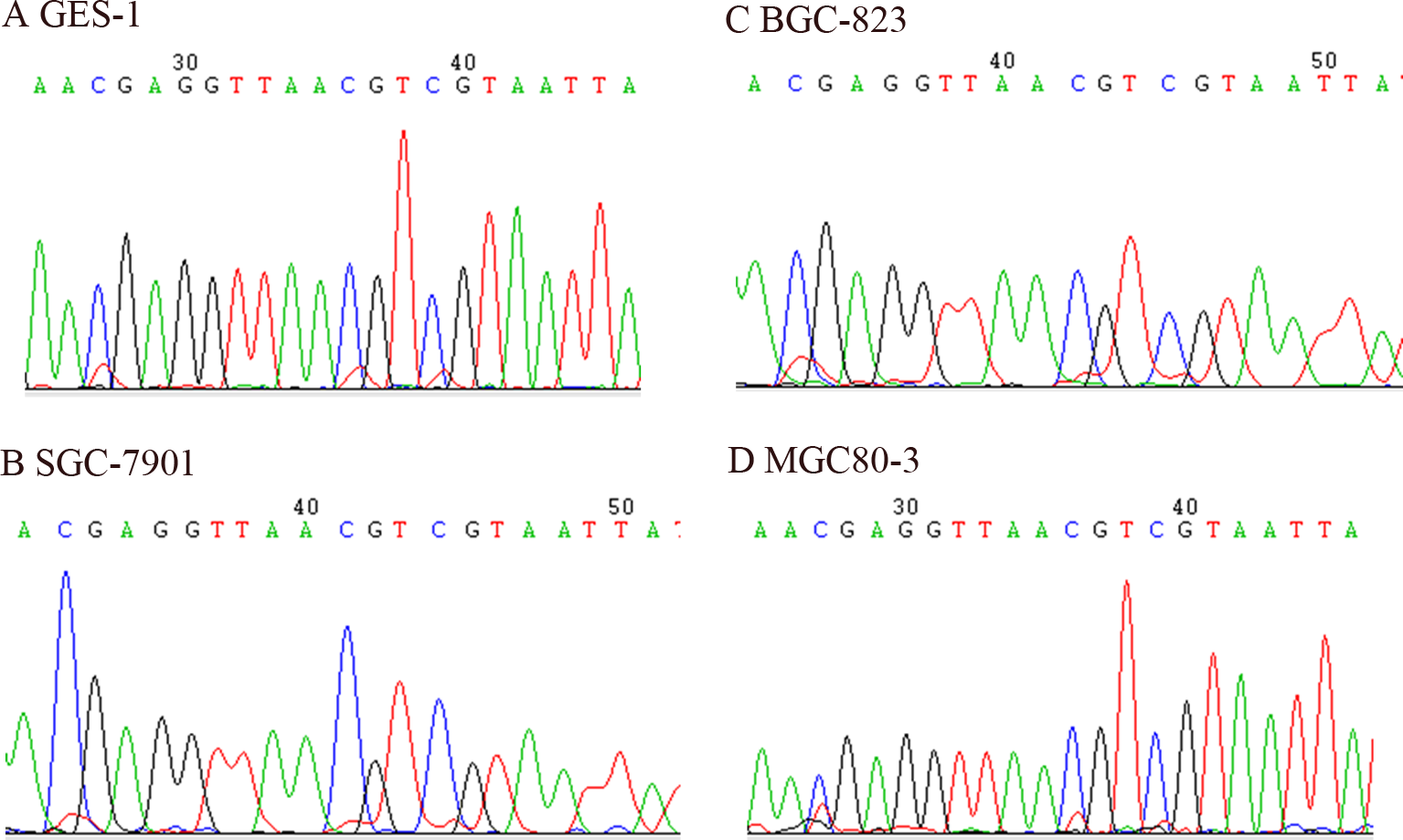

Supplement: Additional file 3: Figure S2. — Methylation status of CDH1 exon 9 in human gastric mucosal epithelial cell line GES-1 and the GC cell lines SGC-7901, BGC-823 and MGC80-3. DNA isolated from cells shows a high C content at all CpGs attributable to reduced bisulfite modification because of nearly complete methylation of the DNA. (TIF 1354 kb) [file 12885_2015_1983_MOESM3_ESM.tif]

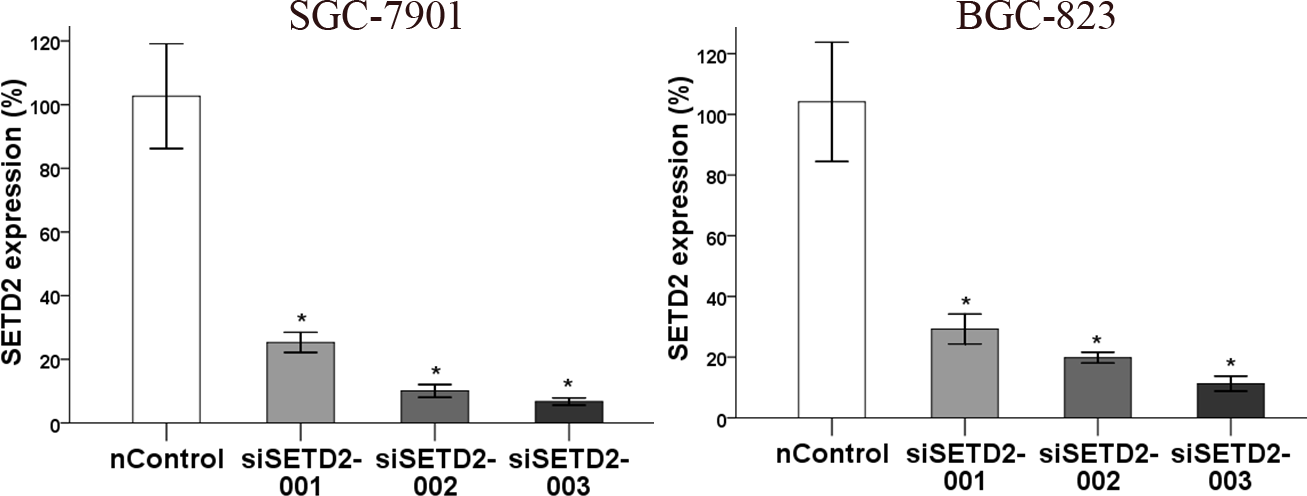

Supplement: Additional file 4: Figure S3. — Interference efficiency of the three kinds of siSETD2 in SGC-7901 (A) and BGC-823 (B) cells. The values were calculated as 2-ΔCt (SETD2-actin). The star * means P < 0.05, indicating statistically significant. (TIF 147 kb) [file 12885_2015_1983_MOESM4_ESM.tif]

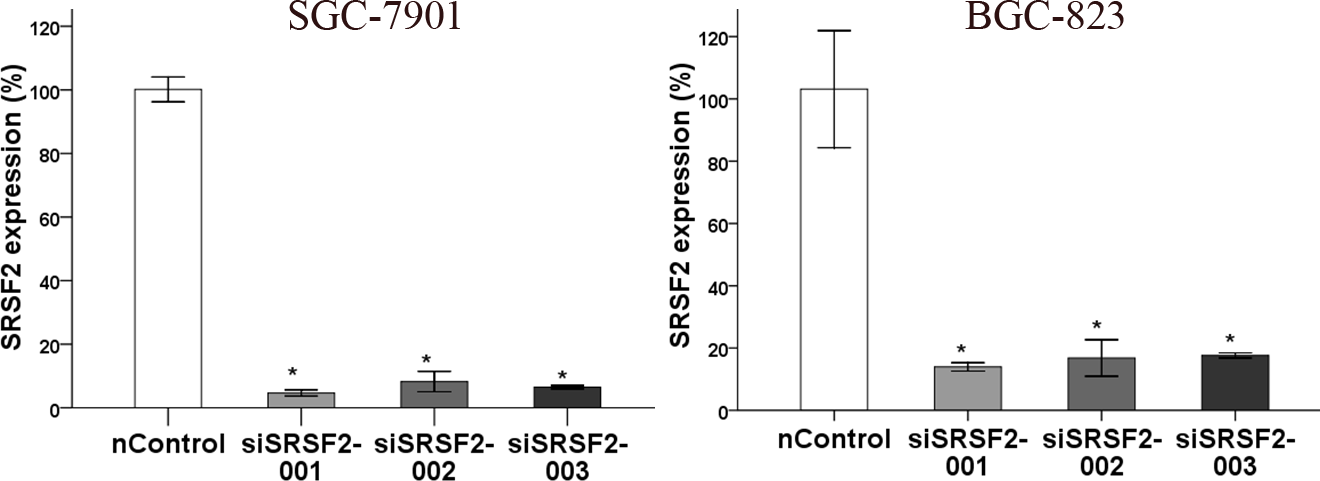

Supplement: Additional file 5: Figure S4. — Interference efficiency of the three kinds of siSRSF2 in SGC-7901 (A) and BGC-823 (B) cells. The values were calculated as 2-ΔCt (SRSF2-actin). The star * means P < 0.05, indicating statistically significant. (TIF 147 kb) [file 12885_2015_1983_MOESM5_ESM.tif]
